# The Anemia Risk Warning Model Based on a Noninvasive Method: Key Insights and Clarifications

**DOI:** 10.2196/73297

**Published:** 2025-04-22

**Authors:** Jiaqi Wei, Nana Zheng, Depei Wu

**Affiliations:** 1 Jiangsu Institute of Hematology National Clinical Research Center for Hematologic Diseases The First Affiliated Hospital of Soochow University Suzhou China; 2 Institute of Blood and Marrow Transplantation Collaborative Innovation Center of Hematology Soochow University Suzhou China

**Keywords:** anemia, hemoglobin, spectroscopy, machine learning, risk warning model, Shapley Additive Explanation

We recently read with great interest the article by Zhang et al [1], which presents a noninvasive technique for diagnosing anemia using facial visible light reflectance spectroscopy, combined with machine learning (ML) algorithms for predictive modeling. Anemia is a widespread public health issue affecting over 1.7 billion people, with symptoms including fatigue and cognitive decline [2]. The study focuses on using facial spectral data to develop a risk warning model for anemia, providing a reliable alternative to conventional methods and holding promise for future clinical applications in noninvasive anemia diagnosis. Having perused the article, we wish to recommend some insights and clarifications to enhance the robustness of the results.

Anemia is affected by various factors, including physiological, nutritional, genetic, environmental, and socioeconomic factors. Zhang et al [1] applied stringent matching criteria for age, location, and gender. However, other factors, such as smoking and nutrient intake, can also impact anemia severity, which should be taken into careful consideration. Additionally, skin types in various regions and lighting conditions at different times can also affect spectral reflectance measurements. When light enters human skin, the epidermis and dermis along with the light-absorbing chromophores, such as water, lipid, hemoglobin, and melanin, constitute the scattering medium and determine the skin’s spectral reflectance [3]. Thus, individual variations in skin moisture, temperature, and melanin levels can also significantly alter reflectance. To minimize these effects, standardized protocols for controlling environmental factors should be strictly enforced, and data from a broader range of skin types could be incorporated to enhance the generalizability of the results.

Application of ML methods in medical fields aims to enhance the reliability and predictability of disease diagnosis for future development. Zhang et al [1] used 10 ML algorithms to thoroughly analyze the classifiers, with the support vector machine (SVM) algorithm demonstrating the best performance. However, despite its superior performance, the SVM model may still be prone to overfitting due to the relatively small sample size. Additionally, the lack of external validation using independent datasets raises concerns about the model’s actual performance. Future studies should incorporate cross-validation with larger datasets to enhance predictive accuracy and generalizability.

Noninvasive diagnostic methods are currently advancing rapidly. By analyzing facial light reflectance, this study pinpoints specific facial regions and wavelengths strongly correlated with anemia risk. However, it would be beneficial to compare its diagnostic performance with that of other noninvasive hemoglobin measurements, such as the microwave resonant system [4] and photoplethysmography signal sensors [5]. A comparative study could help assess whether spectral reflectance provides higher accuracy and clinical utility than these methods or when combined with them.

In conclusion, the study identifies significant differences in the facial spectral characteristics of patients with anemia and develops a high-accuracy warning model for predicting anemia risk. This noninvasive technique provides valuable opportunities to analyze facial signs of anemia and offers a more affordable approach to facilitate diagnosis. Undoubtedly, this research reveals a promising direction for personalized patient management and lays the foundation for potential applications in community-based health screenings.

## Abbreviations

ML: machine learning
SVM: support vector machine
